# COVID-19 and Tweets About Quitting Cigarette Smoking: Topic Model Analysis of Twitter Posts 2018-2020

**DOI:** 10.2196/36215

**Published:** 2022-05-16

**Authors:** J Lee Westmaas, Matthew Masters, Priti Bandi, Anuja Majmundar, Samuel Asare, W Ryan Diver

**Affiliations:** 1 Population Science Department American Cancer Society Kennesaw, GA United States

**Keywords:** COVID-19, machine learning, pandemic, quit smoking, topic model analysis, Twitter, social media, smoking cessation, latent Dirichlet allocation, tweet, public health

## Abstract

**Background:**

The risk of infection and severity of illness by SARS-CoV-2 infection is elevated for people who smoke cigarettes and may motivate quitting. Organic public conversations on Twitter about quitting smoking could provide insight into quitting motivations or behaviors associated with the pandemic.

**Objective:**

This study explored key topics of conversation about quitting cigarette smoking and examined their trajectory during 2018-2020.

**Methods:**

Topic model analysis with latent Dirichlet allocation (LDA) identified themes in US tweets with the term “quit smoking.” The model was trained on posts from 2018 and was then applied to tweets posted in 2019 and 2020. Analysis of variance and follow-up pairwise tests were used to compare the daily frequency of tweets within and across years by quarter.

**Results:**

The mean numbers of daily tweets on quitting smoking in 2018, 2019, and 2020 were 133 (SD 36.2), 145 (SD 69.4), and 127 (SD 32.6), respectively. Six topics were extracted: (1) need to quit, (2) personal experiences, (3) electronic cigarettes (e-cigarettes), (4) advice/success, (5) quitting as a component of general health behavior change, and (6) clinics/services. Overall, the pandemic was not associated with changes in posts about quitting; instead, New Year’s resolutions and the 2019 e-cigarette or vaping use–associated lung injury (EVALI) epidemic were more plausible explanations for observed changes within and across years. Fewer second-quarter posts in 2020 for the topic e-cigarettes may reflect lower pandemic-related quitting interest, whereas fourth-quarter increases in 2020 for other topics pointed to a late-year upswing.

**Conclusions:**

Twitter posts suggest that the pandemic did not generate greater interest in quitting smoking, but possibly a decrease in motivation when the rate of infections was increasing in the second quarter of 2020. Public health authorities may wish to craft messages for specific Twitter audiences (eg, using hashtags) to motivate quitting during pandemics.

## Introduction

### Background

Researchers and health authorities (eg, Centers for Disease Control and Prevention) are increasingly using Twitter, a social media platform with over 35 million daily active users in the United States [1], to achieve public health goals. These include disseminating health information and surveillance or prediction of health-related behaviors [2-5]. Tobacco researchers have also investigated Twitter postings (tweets) to identify and monitor attitudes or behaviors of people who smoke cigarettes or other tobacco products [6-11]. The aim of this study was to examine key topics of public conversations about quitting cigarette smoking during the COVID-19 pandemic. This could help determine whether public health action(s) to address smoking or quitting during a pandemic may be warranted.

There are several reasons why the COVID-19 pandemic may have influenced cigarette smoking behavior. Perceiving oneself at heightened risk for disease due to smoking can trigger attempts to quit that lead to abstinence [12,13]. COVID-19 has caused more than 6 million deaths worldwide [14], and is understood to be a respiratory disease for which the risk of infection and severity of illness are significantly elevated for cigarette smokers (due to preexisting damage to the respiratory system) [15,16]. Rates of quitting smoking would be expected to increase during the pandemic, at least to the extent that people who smoke are aware of their elevated risk. Yet, analyses from the North American Quitline Consortium found a 27% reduction in calls to quitlines in 2020 for cessation counseling compared to those in 2019 [17]. The largest decreases occurred in the second (–39%) and third (–30%) quarters of 2020, paralleling the onset and unfolding of the pandemic. These data suggest that people who might otherwise have quit continued smoking instead (ie, postponed or canceled plans to quit). However, given the very low reach of quitlines [18,19], it is unclear whether smokers who intended to call quitlines for cessation assistance are representative of the overall population of people who smoke.

Although perceiving oneself at higher risk of infection or illness might increase the motivation to quit smoking for some individuals, for many individuals the pandemic may have had the opposite effect. Several studies have documented increased anxiety or depression linked to the pandemic [20-27], and for many who smoke, negative affect or stress is a trigger of cravings and/or smoking [28-31]. At its peak, the pandemic had, and continues to have, multiple stressful sequelae (eg, on employment, income, social interactions, health care, child care) [32], which may have led to cigarette smoking as a stress-relief strategy. Moreover, lockdowns that necessitated working from home could have obviated workplace smoking restrictions that had previously limited consumption. Using cigarettes to cope with stress, or the absence of restrictions on smoking, may partly explain findings from data collected by the Alcohol and Tobacco Tax and Trade Bureau of the US Department of Treasury. Analyses of these data indicated that sales of cigarettes to retail and wholesale outlets increased during 2020, a reversal from annual decreases since 2015 [33].

Studies that have directly asked smokers about their smoking patterns or quitting behaviors during the pandemic have been inconclusive, with some reporting increases, decreases, or both. One cross-sectional study of 366 mostly dual users of cigarettes and electronic cigarettes (e-cigarettes) conducted early in the pandemic in the United States (April 2020) found that approximately half reported no change in their motivation to quit due to COVID-19, with approximately one-third reporting an increase. Approximately one-fifth of the sample reported trying to quit because of COVID-19 [34].

An online survey (also conducted early in the pandemic) of 6800 cigarette smokers from 5 countries found that although 41% and 37% of US and UK smokers, respectively, said that they considered quitting, only 27% (US smokers) and 21% (UK smokers) reported actually making a quit attempt [35]; however, since the respondents were asked only about recent quit attempts, quitting for reasons other than COVID-19 could not be ruled out [35]. A qualitative study conducted in April-May 2020 of 44 individuals who used either cigarettes or electronic nicotine delivery systems found increased consumption to be more common, but also noted a decrease among “social” users [36].

Overall, while several studies have suggested that smoking intensity increased during the pandemic [37-42], there have been exceptions [43,44]. Methodological differences across studies likely account for varying results in terms of design (eg, longitudinal vs cross-sectional), sample size, measures used, geographic location, sociodemographic differences (eg, age, gender), or other factors (eg, exposure to news media coverage of the pandemic that could exacerbate stress reactions [45]).

### Current Focus

We propose that a topic model analysis of tweets related to quitting cigarette smoking in 2020 and prior years could illuminate how the pandemic influenced thoughts about, plans, or attempts to quit. Since Twitter posts are user-generated, analyses of postings represent an organic and real-time approach compared to investigator-initiated surveys, which involve lags in development, administration to respondents, and data collection and analysis. The frequency of tweets can also be examined over many days, weeks, or even years to gain insights into motivations and trends. Other appealing features of Twitter include its use by approximately one quarter of US adults, with similar proportions of white, Black, or Hispanic users [46-48].

Prior work also suggests links between talking about tobacco-related topics on Twitter and subsequent tobacco use behaviors [49]. Additionally, Twitter conversations are sensitive to ongoing events and are useful for monitoring health-related trends [2]. As such, tracking the trajectory (frequency) of key topics of conversations pertaining to quitting cigarette smoking during and prior to the pandemic could offer insights about its relationship to quitting behaviors. Findings from this research could potentially provide early warning signs for public health interventions to address adverse effects of the pandemic using Twitter or other social media platforms.

## Methods

### Overview

We used a latent Dirichlet allocation (LDA) model to identify dominant topics among Twitter postings that included the phrase “quit smoking.” LDA, a form of unsupervised machine learning used to classify documents [50], assumes that each document (in this case a tweet) may belong to more than one topic. Words from tweets are randomly placed into topics but are systematically moved to different topics so that “fit” can be iteratively tested. After a number of iterations, “topics” emerge consisting of a combination of words with associated “weights.” The combination of words provides insight into the theme of a topic. Tweets with the highest probability of belonging to a particular topic are considered to be most representative of that topic.

By using LDA to identify topics associated with the term “quit smoking” and examining their frequency in 2018, 2019, and 2020, we aimed to (1) identify the most important themes in natural discussions about quitting cigarettes, and (2) their changes in frequency in 2020 in the context of the pandemic.

### Ethics Approval

Institutional review board approval was not required as our data collection involved aggregate analysis of visible public data without linking of personal identifiers. Twitter’s user consent agreement also includes the possibility of tweets being used in research.

### Data Collection

All (100%) daily tweets containing the phrase “quit smoking” posted between January 1, 2018, and December 31, 2020, were collected using Social Studio, an online social media engagement platform owned by Salesforce. This resulted in a total of 201,181 tweets.

Tweets were geographically limited to US accounts to increase generalizability to the US population and because of differences among countries as to when public health measures were implemented to reduce the pandemic’s spread.

Only tweets containing the phrase “quit smoking” were collected. We had originally employed an expansive list of search terms to capture quitting smoking, but this resulted in poorer topic modeling due to the colloquial nature of tweets (eg, using the search phrase “stop smoking” was often used pejoratively in replies to tweets unrelated to tobacco use).

Retweets, quote tweets, and identical tweets were removed from the data set to reduce spam from bots associated with products and website promotion or activity. To reduce noise related to cannabis use, tweets containing the words “weed,” “blunt,” “crack,” “roach,” “baked,” “dabs,” “marijuana,” “bong,” “mary jane,” “maryjane,” and “pot” were removed [51].

To improve model performance, all tweets were converted to lower case and URLs were removed. Tweets were converted into lists of individual words (ie, tokenization). Individual words were then tagged with their part of speech (eg, “verb” or “noun”) and shortened to their stem. This conversion of words such as “coughing” or “coughed” to simply “cough” prevents misclassification due to tense or plurality. Words that contained only digits were removed because they do not provide meaningful information to a topic model. Stop words; words such as “the,” “to,” or “and”; as well as words less than two characters long were removed to prevent inclusion of noninformative words in the topic model. Two-word phrases that occurred in at least 20 tweets were combined into one “word” so that commonly related words would be placed in a topic together.

### Topic Selection

For topic modeling, we used the gensim library for Python, which provides an LDA model creation function and has a sizeable documentation library and large feature set. Model parameters such as the document-topic distributions (*α*) and topic-word distributions (*η*) can also be automatically determined.

A (bigram) model was first trained on tweets from 2018 and then applied to tweets posted in 2019 and 2020. We used the 2018 tweets as the training set to identify topics related to cessation that would not be influenced by COVID-19–related topics and that would not be present in our comparison data set (2019).

To determine the total number of topics in the 2018 data, the model was run for 10 passes and 200 iterations. For the first iteration, the total number of topics was set to 2 and the model’s coherence statistic was noted. This process was repeated progressively for up to 50 topics, and the resulting coherence scores were graphed to enable selection of the optimal number of topics. The LDA model also indicated the proportionate distribution among final topics for each tweet (eg, a tweet could have a proportion of 0.75 for one topic and 0.25 for a second topic if the tweet broached both topics). Final topics were labeled by two authors (JLW and MM) based on the most influential words for that topic and by examining representative tweets. For each final topic, proportions were summed to derive the total number of tweets belonging to that topic.

### Statistical Analysis

For each topic, we analyzed mean daily tweets by quarters because rates of infection in the second quarter of 2020 had begun to rise more steeply compared to those in the previous 3 months (first quarter) of the year [52]. Analysis of variance and follow-up pairwise comparisons (with Bonferroni correction) compared mean numbers of daily tweets among quarters within each year. We also compared, by quarter, pairwise differences across years (eg, January-March for 2020, 2019, and 2018). As a measure of the sensitivity of topics to the pandemic, we examined the proportion of each topic’s tweets in 2020 (by quarter) that mentioned “coronavirus” or “covid.”

## Results

### Overview of Topics Identified and Trends Over Time

The average daily number of tweets related to quitting smoking in 2018, 2019, and 2020 were 133 (SD 36.2), 145 (SD 69.4), and 127 (SD 32.6), respectively. Ten topics were initially identified as optimal. Of these, four were clearly spam or advertisements and were eliminated, resulting in six final topics that were selected for analyses: (1) *need to quit*, (2) *personal experiences*, (3) *e-cigarettes*, (4) *advice/success*, (5) *quitting as a component of general health behavior change,* and (6) *clinics/services.* The number of quit smoking–related tweets, by topic and quarter, are provided in Table 1. Examples of tweets from each topic appear in Table 2.

**Table 1:** Number and percent of “quit smoking” tweets by topic and year for each quarter.

Topic		2018			2019			2020	
		N	n (%)	N		n (%)	N		n (%)
**Need to quit**									
	January-March	13,839	1654 (11.95)	13,709		1382 (10.08)	13,732		1311 (9.55)
	April-June	11,971	1503 (12.56)	10,696		1146 (10.71)	10,605		1121 (10.57)
	July-September	11,206	1466 (13.08)	15,178		1402 (9.24)	10,608		1083 (10.21)
	October-December	11,456	1262 (11.02)	13,433		1202 (8.95)	11,683		1191 (10.19)
**Personal experiences**									
	January-March	13,839	3610 (26.09)	13,709		3646 (26.60)	13,732		3641 (26.52)
	April-June	11,971	3185 (26.61)	10,696		2887 (26.99)	10,605		2895 (27.30)
	July-September	11,206	2934 (26.18)	15,178		3624 (23.88)	10,608		3001 (28.29)
	October-December	11,456	2898 (25.30)	13,433		2887 (21.49)	11,683		3313 (28.36)
**Electronic cigarettes**									
	January-March	13,839	2447 (17.68)	13,709		2586 (18.86)	13,732		2671 (19.45)
	April-June	11,971	2006 (16.76)	10,696		1896 (17.73)	10,605		1617 (15.25)
	July-September	11,206	1729 (15.44)	15,178		4222 (27.82)	10,608		1594 (15.03)
	October-December	11,456	2012 (17.56)	13,433		3483 (25.93)	11,683		1573 (13.46)
**Advice/success**									
	January-March	13,839	1767 (12.77)	13,709		1857 (13.55)	13,732		2108 (15.35)
	April-June	11,971	1569 (13.11)	10,696		1560 (14.58)	10,605		1651 (15.57)
	July-September	11,206	1549 (13.82)	15,178		2186 (14.40)	10,608		1619 (15.26)
	October-December	11,456	1548 (13.51)	13,433		2000 (14.89)	11,683		1837 (15.72)
**Health changes**									
	January-March	13,839	2761 (19.95)	13,709		2860 (20.86)	13,732		2759 (20.09)
	April-June	11,971	2577 (21.53)	10,696		2291 (21.42)	10,605		2317 (21.85)
	July-September	11,206	2426 (21.65)	15,178		2728 (17.97)	10,608		2319 (21.86)
	October-December	11,456	2476 (21.61)	13,433		2464 (18.34)	11,683		2485 (21.27)
**Clinics/services**									
	January-March	13,839	1600 (11.56)	13,709		1378 (10.05)	13,732		1242 (9.04)
	April-June	11,971	1131 (9.45)	10,696		917 (8.57)	10,605		1004 (9.47)
	July-September	11,206	1101 (9.83)	15,178		1015 (6.69)	10,608		991 (9.34)
	October-December	11,456	1259 (10.99)	13,433		1085 (8.08)	11,683		1284 (10.99)

**Table 2:** Topics and representative tweets.

Topic	Key words	Sample tweets
Need to quit	need, really, gotta, cigarette, wanna, help, like, know, stop, friend, pregnant, sad, job, real, die, hard, soon, damn, win, cigs	“I need to quit smoking. tobacco is a demon it’s so addictive I gotta stop. I threw out my cigarettes just now. I want them tho.” “I need to quit smoking man. This shit has taken away my life. But there isn’t shit to do in Lorain besides rolling up with your friends.”
Personal experiences	cigarette, year, day, since, time, year_ago, last, try, month, week, buy, still, gonna, decide, hard, life, cold_turkey, ago, smell, feel	“I quit smoking right around this time of year, 10 years ago (passover). After the first year the cravings stopped, pretty much completely. Yet all of a sudden, a minute ago I got the strongest urge, even visualized lighting up. I guess it never does go all the way away?” “I’ve decided to quit smoking. For good. Hopefully. I hadn't smoked since Saturday. I bought a pack tonight. Got home. Opened the pack. Pulled out a cig. And was about to light it when I stopped. I ran to the trash can, threw the lighter, cig and the whole pack away.”
E-cigarettes	help, vaping, people, use, want, vape, tobacco, start, nicotine, flavor, product, try, may, health, find, juul, offer, adult, learn, good	“I know of hundreds of adults that have quit smoking cigarettes in favor of candy flavored ejuice. I find it hard to believe that children are buying this in a large scale, and sounds like a parental and education problem rather than a candy flavor problem. this creates false fear” “With vaping, you take away most of the harmful parts of tobacco. I'll admit the science is not fully developed, esp on negative consequences, but it seems clear that vaping helps people quit smoking and is safer than cigarettes.”
Advice/success	would, help, say, never, use, life, people, year, tell, wish, love, work, give, change, mom, doctor, easy, addiction, still, nicotine	“I can tell you how to quit smoking and it won't suck as much. Get the patches use the highest dosage for 21 days that’s how long it’ll take to break the hand-to-mouth habit after 21 days get a lesser dosage after 21 days of. step down again. It works” “Had some bad days in 2018. made big changes! down 40lbs, quit smoking > 3 mths, walking 5 mi. a day. and yes, the other stuff too- but much longer! thank you! you could've been mean, but all choose not to! thankful for the ass kicking I needed and the silver lining I found!”
Quitting as a component of general health behavior change	get, good, want, like, think, year_ago, drinking, drink, cigarette, eat, feel, start, work, life, try, job, people, stop, since, never	“Life is good. My daughter is smart, healthy, witty, & beautiful. I have a stable job that I’m good at & don’t hate. I’m about to trade in my 2010 Malibu & get my own place. My credit score is bomb. I quit smoking cigarettes almost a year ago. I’m super blessed.” “So far this year I have stopped eating fried food, started getting up earlier, started eating more vegetarian/vegan food types, and quit smoking cigarettes. I intend to constantly peak in 2018.”
Clinics/services	free, help, get, call, time, support, good, today, stop, need, health, reason, ready, visit, plan, book, therapy, service, contact, register	“Pregnant and ready to quit smoking? our baby & me - tobacco free program can help! enroll today to start your quit journey and start earning free diapers and baby gear! Call to schedule your first appointment. #pregnancy #smoking #smokefree #baby #free #health” “Happy National Non-Smoking Week! #mlhu is hosting a quit smoking workshop in #ldnont Thursday, January 25. participants will receive free nicotine patches & educational material. To see if you’re eligible and to register, please call #nnsw #nnsw2018”

### Need to Quit

**Figure 1:**
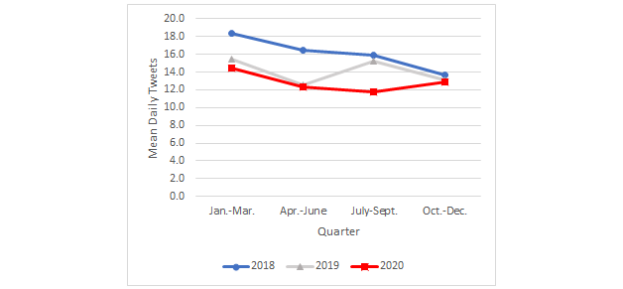
Mean daily tweets about need to quit by year and quarter.

Tweets for which *need to quit* was the dominant topic expressed a desire or need to quit smoking cigarettes in the present or near future (eg, “I’m going to really try to stop smoking after this weekend”; see Table 2 for additional examples of tweets from each topic). In 2020, the mean daily frequency of tweets about the need to quit smoking (Figure 1) was higher in the first quarter (mean 14.4, SD 3.6) compared to the second (mean 12.3, SD 2.6; *P*=.001), third (mean 11.8, SD 2.7; *P*=.001), and fourth (mean 12.9, SD 3.5; *P*=.009) quarters; the last 3 quarters of 2020 did not differ significantly from each other.

The frequency of tweets about the need to quit was significantly higher for the first 3 quarters of 2018 compared with those at the same periods in 2020 (mean 18.4 vs 14.4, *P*=.001; 16.5 vs 12.3, *P*=.001; 15.9 vs 11.8, *P=*.001). From October to December, however, there were no significant differences across all 3 years in daily tweets about the need to quit. The only significant difference between 2019 and 2020 was observed for the 3rd quarter (mean 15.2 vs 11.8, respectively; *P=*.001).

### Personal Experiences

**Figure 2:**
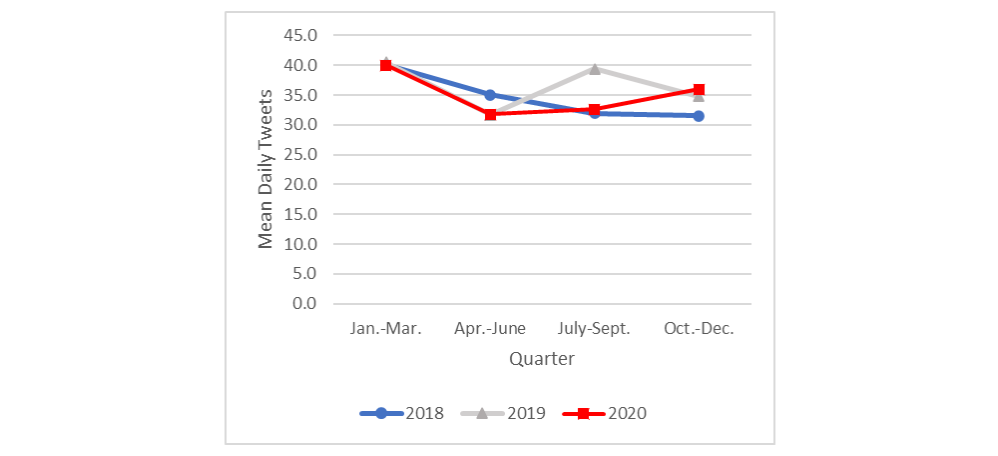
Mean daily tweets about personal experiences by year and quarter.

Tweets for which *personal experiences* was the dominant topic expressed personal actions or difficulties in attempting to quit or positive effects of having quit (eg, “I’m trying to stop smoking but I’m dying to smoke”). In 2020, the mean daily frequency of tweets about personal experiences with quitting (Figure 2) was higher in the first quarter (mean 40.0, SD 11.3) compared to the second (mean 31.8, SD 6.9; *P=*.001), third (mean 32.6, SD 6.2; *P=*.001), and fourth (mean 36.0, SD 10.5; *P=*.02) quarters. Of the 4 quarters in 2020, the frequency of the last quarter’s tweets was the second highest and significantly greater than that for the second quarter of 2020 (*P=*.01), suggesting an uptick in frequency late in 2020. The mean number of daily tweets in the last quarter of 2020 (mean 36.0, SD 10.5) was also significantly greater than that for the same quarter in 2018 (mean 31.5, SD 8.7; *P*=.005), but not compared to that in 2019 (mean 34.8, SD 9.5; *P*=.99).

A substantial spike in tweets about personal experiences with quitting was observed in 2019 during the third quarter, coincident with the e-cigarette or vaping product use–associated lung injury (EVALI) epidemic. The mean number of daily tweets during the third quarter in 2019 was significantly higher (mean 39.4, SD 18.5) than that in the third quarter of 2018 (mean 31.9, SD 6.6; *P=*.001) or in 2020 (mean 32.6, SD 6.2; *P=*.001).

### E-cigarettes

**Figure 3:**
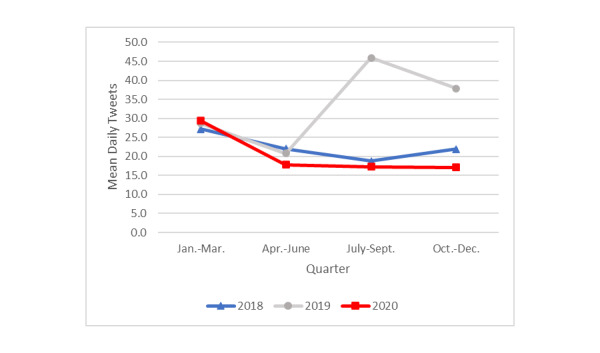
Mean daily tweets about electronic cigarettes by year and quarter.

Tweets for which *e-cigarettes* was the dominant topic referred to e-cigarettes in the context of quitting smoking (eg, “…finally decided to buy a JUUL to quit smoking cigarettes, but I paid $50 for a starter kit with only two pods…four were advertised online.. not good”). In the first quarter of 2020, the mean number of daily tweets about e-cigarettes (Figure 3) was significantly higher (mean 29.3, SD 14.6) compared with those in the second (mean 17.8, SD 6.2; *P*=.001), third (mean 17.3, SD 4.8; *P*=.001), and fourth (mean 17.1, SD 6.5; *P*=.001) quarters of 2020. For these last 3 quarters in 2020, the mean daily number of tweets about e-cigarettes did not differ significantly from each other. Cross-year comparisons indicated that the mean number of fourth-quarter tweets in 2020 was significantly lower (mean 17.1, SD 6.5) than those in the same period in 2019 (mean 37.9, SD 15.3; *P=*.001) and 2018 (mean 21.9, SD 11.6; *P=*.02).

The pattern of tweets about e-cigarettes across quarters in 2018 was similar to that of 2020 (a drop in frequency of tweets after the first quarter, and similar levels in subsequent quarters). In 2019, there was a substantial spike in the third quarter (mean 45.9, SD 61.2) compared to the first (*P*=.003) and second (*P=*.001) quarters that likely reflected heightened awareness and discussion about the role of e-cigarettes in causing EVALI. This heightened frequency of tweets in the third quarter of 2019 continued into its final quarter (mean 37.9, SD 15.3).

### Advice/Success

**Figure 4:**
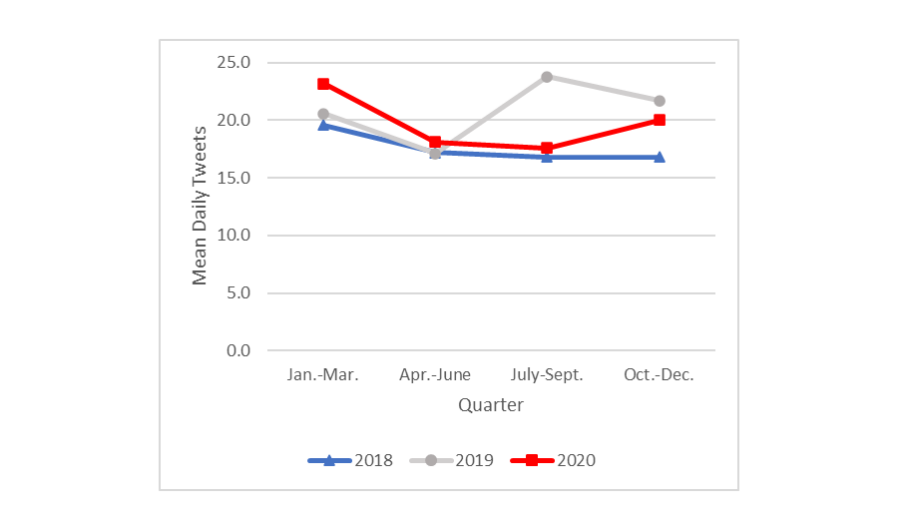
Mean daily tweets about advice/success by year and quarter.

Tweets for which *advice/success* was the dominant topic expressed personal success in having quit smoking and/or provided encouragement or advice for quitting (eg, “That’s when you get help for your addiction to quit smoking…”). The mean number of daily tweets with quitting advice/success stories (Figure 4) was significantly higher in the first quarter of 2020 (mean 23.2, SD 7.1) compared with those in the second (mean 18.1, SD 5.3; *P*=.001), third (mean 17.6, SD 3.7; *P*=.001), and fourth (mean 20.0, SD 5.4; *P=*.001) quarters. First-quarter tweets in 2020 were also significantly higher compared with those in the same period in 2019 (mean 20.6, SD 6.1; *P=*.03) and 2018 (mean 19.6, SD 6.3; *P*=.001). Fourth-quarter tweets in 2020 (mean 20.0, SD 5.4) were significantly higher compared with those in the same period in 2018 (mean 16.8, SD 8.9; *P*=.008), but did not differ from those of 2019 (mean 21.7, SD 6.0; *P=*.26).

In 2019, the third quarter’s mean daily number of tweets with quitting advice/success stories was significantly elevated (mean 23.8, SD 18.3) compared with that of the previous quarter (mean 17.1, SD 4.5; *P=*.001). This heightened level of tweets in the third quarter of 2019 continued into its final quarter (mean 21.7, SD 6.0).

### Quitting as a Component of General Health Behavior Change

Tweets for which *quitting as a component of health behavior change* referenced health behavior change in general that also included quitting smoking, for example:

Remembering how important it is to have self control has: 1. Helped me quit smoking cigarettes 2. Realize the control alcohol had over me when I was weak 3. Made me a better friend 4. Be optimisitic about my overall self worth 5. Be a better worker at my job.

**Figure 5:**
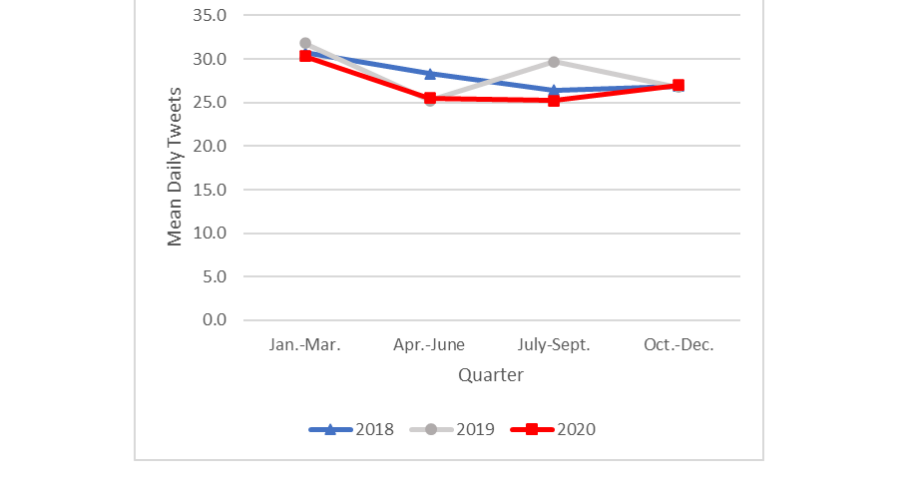
Mean daily tweets about quitting as a component of general health behavior change by year and quarter.

In 2020, the mean daily frequency of tweets about quitting in the context of health behavior change (Figure 5) was significantly higher in the first quarter (mean 30.3, SD 7.9) compared with those in the second (mean 25.5, SD 5.4; *P=*.001), third (mean 25.2, SD 5.4; *P=*.001), and fourth (mean 27.0, SD 6.2; *P*=.003) quarters.

The mean number of daily tweets was also lower in the second quarter of 2020 (mean 25.5, SD 5.4) compared with that in the same period in 2018 (mean 28.3, SD 6.6; *P*=.002), although not compared with that in 2019 (mean 25.2, SD 5.1; *P*=.99). The frequency of tweets about quitting in the context of general health behavior change was lower in the third quarter of 2020 (mean 25.2, SD 5.4) compared with that in the same period in 2019 (mean 29.7, SD 13.3; *P*=.002), but not compared to 2018 (mean 26.4, SD 4.9; *P*=.99).

### Clinics/Services

Tweets for which *clinics/services* was the dominant topic offered help for quitting by clinics, companies, or support services, for example:

Quit smoking for the first 7 days of the month and you could win $500! Why is the first week so important? Because if you are successful for the first week, you’re 9x more likely to quit for good. And that’s amazing!

**Figure 6:**
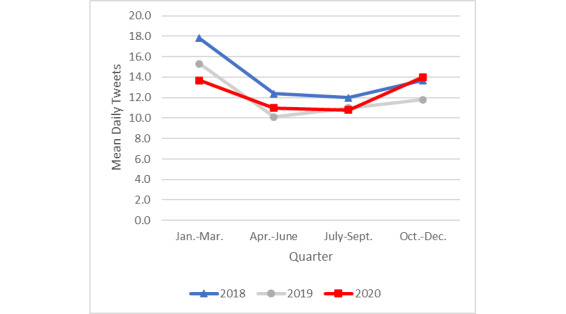
Mean daily tweets about clinics/services by year and quarter.

The general pattern for frequency of tweets about support services for quitting was similar across quarters for all 3 years: higher in the first quarter, followed by a decrease in the second quarter that was maintained in the third quarter, followed by modest increases in the last quarter of the year (Figure 6).

For 2020, the frequency of daily tweets about support services for quitting was higher in the first quarter (mean 13.6, SD 5.1) compared with those in the second (mean 11.0, SD 3.6; *P*=.002) and third (mean 10.8, SD 3.7; *P*=.001) quarters, but not compared with that of the fourth quarter (mean 14.0, SD 6.5, *P*=.99) of 2020. In the last quarter of 2020, however, the increase in tweets for support services was significantly greater compared with those in the previous two quarters of 2020 (both *P=*.001). The mean frequency of tweets about support services for the first quarter of 2020 was significantly lower (mean 13.6, SD 5.1) than that of the first quarter of 2018 (mean 17.8, SD 7.4; *P=*.001).

### Proportion of Tweets on Each Topic Including “Coronavirus” or “Covid”

**Figure 7:**
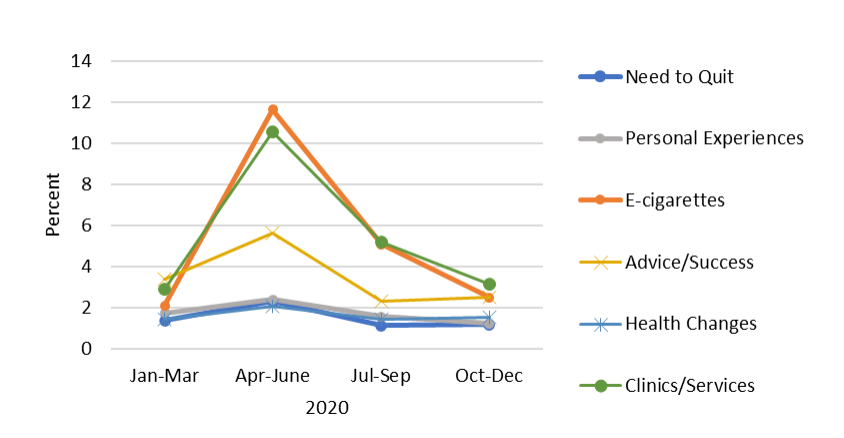
Percentage of tweets for each topic in 2020 that mentioned "coronavirus" or "covid," by quarter.

In 2020, for all topics, there were observable increases in the second quarter in the proportion of tweets for each topic that mentioned “coronavirus” or “covid” (Figure 7). The largest absolute increase was observed for e-cigarettes; in the second quarter of 2020, the proportion of tweets about e-cigarettes in the context of quitting smoking that included “coronavirus” or “covid” increased from 2.1% to 11.7%. By the third quarter, however, the proportion had returned to close to first-quarter levels. This pattern was evident for all topics but was the greatest for *e-cigarettes*, *clinics/services*, and *advice/success*.

## Discussion

### Principal Findings

This study used topic model analysis to observe if and/or how conversations, attitudes, or behavior related to quitting cigarettes changed before and during the pandemic. Six topics were identified by our topic model analysis, all of which would appear to be valid indicators of attitudes, plans, or behaviors regarding quitting cigarettes. Topics were (1) *need to quit*, (2) *personal experiences*, (3) *e-cigarettes*, (4) *advice/success*, (5) *quitting as a component of general health behavior change,* and (6) *clinics/services.* Moreover, our topics were sensitive to the effects of the pandemic, judging from increases in the second quarter of 2020 in the proportion of tweets on each topic that included the terms “coronavirus” or “covid.” In addition, we observed a striking increase in 2019 in tweets related to e-cigarettes and quitting that coincided with the EVALI outbreak, illustrating how external events can generate relevant and potentially valuable information in real time.

For all topics for 2018, 2019, and 2020, mean daily frequencies of posts were generally the highest in the first quarter of the year compared with those in the second and third quarters. However, the size of the reduction in frequency of posts from the first to the second quarter did not differ in 2020 from those in the two previous years; this suggests (for all 3 years) an effect of New Years’ resolutions having either been achieved by the second quarter or the dissipation of motivation to quit after failed New Year’s quit attempts. Moreover, for the second quarter of 2020, when COVID-19 infection rates were rising sharply, for all topics, there were no significant differences in mean daily frequency of tweets in 2020 compared with those in the same period in 2019.

For 2020, a quadratic shape was evident for all topics (except *e-cigarettes*) because of an uptick in posts from the third to the fourth quarter. Reasons for this are not clear but could include the following: (1) smokers realizing a need to quit (perhaps after having postponed such plans earlier in the year), (2) feeling less stress following positive news about vaccine effectiveness in November that resumed interest in quitting, and/or (3) social media or other quit-smoking campaigns such as the nationwide Great American Smokeout in November. Whatever the reason, these results suggest there may have been pent-up demand toward the end of 2020 for cessation services that public health programs might have been able to address.

Only our results for *e-cigarettes* suggest the possibility of somewhat less interest in 2020 in quitting cigarettes that might be pandemic-related. E-cigarettes have become a popular tool for quitting cigarette smoking according to recent research [53-55], with the proportion of people switching to e-cigarettes to quit smoking (35%) similar to that of using any evidence-based treatment [55]. In 2020, in both the second quarter (when COVID-19 cases were surging in the United States) and in the last quarter, tweets about e-cigarettes were significantly lower compared with those in 2018. This suggested diminished interest in 2020 in either using e-cigarettes for quitting, or (by proxy) for quitting in general. These results comport with research from quitlines suggesting diminished interest in 2020 in quitting cigarettes [17], and with data showing increased cigarette sales in 2020 [33]. 

### Limitations

Our analyses did not permit examination of whether the patterns we observed differed by sociodemographic or other user characteristics, as these are not made available by Twitter for confidentiality and privacy reasons. However, according to research conducted by the Pew Foundation in 2019, the median age of Twitter users is younger (47 years) than that of the US population [56]. Twitter users are also more educated and earn higher incomes [56]. Although variables such as gender or age can be inferred from tweets using specialized algorithms, our primary interest was attempting to understand how discussions about quitting changed during the pandemic on a platform used by millions of US residents.

Crises that occurred during the pandemic related to systemic racism and the political climate may have diverted individuals’ propensity to tweet about the topics we identified. This may have been particularly true for younger individuals who at the same time may be less likely to take concerns about COVID-19 or quitting smoking as seriously as older individuals.

It is also not clear to what extent quit smoking–related tweets were posted by nonsmokers; however, it seems unlikely that nonsmokers would tweet about their need to quit, or about e-cigarettes, in the context of quitting smoking.

Our analyses did not control for possible yearly changes in the number of Twitter users. A decrease from 2019 to 2020 in the number of Twitter users, or in overall tweets, could potentially be a reason for the reduction in the frequency of posts for some topics in 2020 compared to the same period in 2019 (and in some cases compared to 2018); however, the number of “monetizable daily active” Twitter users has been increasing annually, including the years covered by our analyses [57]. Moreover, the fact that the number of “quit smoking” tweets by quarter for some topics *increased* in 2020 argues against our results being due simply to decreases in the number of Twitter users in 2020.

### Future Research

Future research that recruits a nationally representative sample of Twitter users who smoke and who allow their Twitter handles to be followed could help determine how generalizable the results obtained from an analysis of tweets are to the general population of people who smoke. Additionally, while there is evidence for the validity of tweets in predicting health-related behaviors [2], future research could assess relationships between the frequency of quit smoking–related tweets and actual behavioral changes. Including perceptions of risk, and individual and social contextual characteristics in such research could help identify for whom a pandemic or other shared event increases or decreases quitting. Such knowledge could then be used to help public health authorities craft messages for specific audiences (eg, using hashtags) to motivate more quitting. Applying our topic model solution to tweets on an ongoing basis can also provide cancer prevention and other institutions with real-time data on how nationwide campaigns or policies may affect cigarette quitting–related thoughts and behaviors.

### Conclusions

Overall, based on the frequencies of posts related to quitting smoking in 2020, the COVID-19 pandemic had limited associations with conversations about quitting or plans to quit. Differences in frequencies across years appeared to be more easily explained by other events such as (after) effects of New Years’ resolutions or the EVALI epidemic. Results for *e-cigarettes* (which are now widely used as a quitting tool) suggest the possibility that the pandemic may have somewhat decreased motivation (or attempts) to quit, but only during the second quarter of 2020 when infection rates were rising rapidly.

## Abbreviations

e-cigarette: electronic cigarette
EVALI: e-cigarette or vaping product use–associated lung injury
LDA: latent Dirichlet allocation
